# Molecular Landscapes of Tumor Dormancy in Gynecologic Malignancies: From Survival Mechanisms to Precision Interception

**DOI:** 10.1002/advs.77941

**Published:** 2026-10-15

**Authors:** Shih‐Hsuan Cheng, Ren‐Wang Peng, Chang‐Cheng Wu, Chi‐Wen Luo, Emmanuel Naveen Raj, Zhi‐Hong Wen, Tzu‐Hung Hsiao, Chia‐Jung Li

**Affiliations:** ^1^ Department of Obstetrics and Gynecology Kaohsiung Veterans General Hospital Kaohsiung Taiwan; ^2^ Department of General Thoracic Surgery Inselspital Bern University Hospital Bern Switzerland; ^3^ Department of BioMedical Research (DBMR) University of Bern Bern Switzerland; ^4^ Department of Obstetrics and Gynecology Zuoying Armed Forces General Hospital Kaohsiung Taiwan; ^5^ Division of Breast Oncology and Surgery Department of Surgery Kaohsiung Medical University Hospital Kaohsiung Medical University Kaohsiung Taiwan; ^6^ School of Medicine College of Medicine National Sun Yat‐sen University Kaohsiung Taiwan; ^7^ National Museum of Marine Biology & Aquarium Pingtung Taiwan; ^8^ Department of Medical Research Taichung Veterans General Hospital Taichung Taiwan; ^9^ Department of Public Health Fu Jen Catholic University New Taipei City Taiwan; ^10^ Institute of Genomics and Bioinformatics National Chung Hsing University Taichung Taiwan; ^11^ Institute of Biopharmaceutical Sciences National Sun Yat‐sen University Kaohsiung Taiwan; ^12^ Center of General Education Cheng Shiu University Kaohsiung Taiwan

**Keywords:** ferroptosis, gynecologic malignancies, longitudinal liquid biopsy, metabolic rewiring, minimal residual disease, spatial multi‐omics, tumor dormancy

## Abstract

Late recurrence in gynecologic malignancies is associated with tumor dormancy and persistent minimal residual disease (MRD). Although current treatments reduce proliferating tumor burden, residual cells may survive therapy and drive delayed relapse. This Review examines dormancy in ovarian, endometrial, and cervical cancers using entry, maintenance, and awakening as an organizing framework rather than a universally demonstrated sequence. Direct evidence is concentrated in ovarian cancer, whereas evidence in endometrial and cervical cancers remains predominantly indirect. We delineate candidate biological dependencies—including redox control, lipid peroxidation buffering, mitochondrial plasticity, adaptive stress‐response programs, epigenetic and post‐transcriptional plasticity, immune regulation, and extracellular matrix remodeling—rather than established vulnerabilities. On this basis, we evaluate two biologically distinct investigational concepts: suppression of reactivation‐associated signals to prolong clinical latency, and state modulation followed by biomarker‐defined treatment, discussed within established disease‐specific care pathways. Finally, we propose a translational framework combining longitudinal liquid biopsy with spatial and single‐cell profiling for MRD monitoring, biological stratification, and future probabilistic inference of residual‐cell states. Together, these concepts support a shift from reactive relapse treatment toward evidence‐ and risk‐constrained recurrence‐interception research and provide a framework for validating residual‐state biomarkers and therapeutic dependencies in gynecologic malignancies.

## Graphical abstract

This Review outlines the biological framework of entry, maintenance, and awakening in dormant minimal residual disease across gynecologic cancers. By mapping critical biological dependencies—from metabolic plasticity to microenvironment interactions—it evaluates novel interception concepts and presents a translational framework combining single‐cell profiling with liquid biopsy to shift clinical paradigms from reactive treatment to proactive relapse interception.

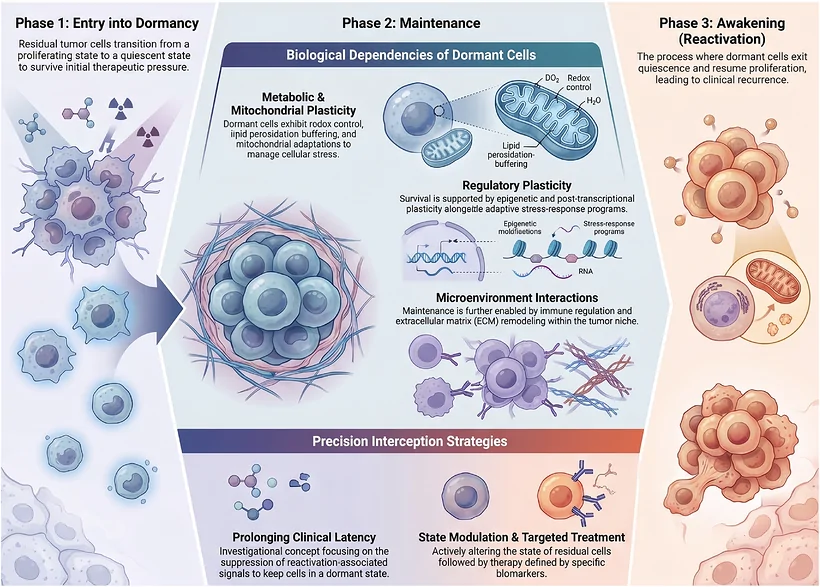

## 1 Introduction

In the clinical management of gynecologic malignancies, including ovarian, endometrial, and cervical cancers, standard treatment strategies—such as maximal cytoreductive surgery, radical hysterectomy, platinum‐based chemotherapy, radiotherapy, or multimodal approaches—often produce substantial initial remission. Despite these early responses, late recurrence remains a leading cause of cancer‐related death. The interval of clinical latency before overt relapse has often been interpreted in relation to tumor dormancy and the persistence of minimal residual disease (MRD), although the two terms describe related but biologically distinct concepts. Historically, tumor dormancy was invoked to explain prolonged intervals between tumor‐cell dissemination and clinically detectable recurrence and was frequently regarded as a passive state of growth arrest caused by inadequate proliferative signaling or limited nutrient availability [1, 2]. More recent primary studies, however, suggest that dormancy is better defined as a dynamic survival state that is actively maintained, marked by epigenetic plasticity and shaped by ongoing interaction with the microenvironment [3, 4, 5].

In this Review, tumor dormancy refers to prolonged and potentially reversible growth restraint of viable tumor cells or microscopic lesions [2, 6]. Cellular quiescence describes a state of low proliferative activity, whereas disseminated tumor cells (DTCs) are defined by their anatomical dissemination and do not necessarily indicate a specific proliferative state [2, 7, 8]. Drug‐tolerant persister cells survive therapeutic exposure but do not necessarily meet the temporal and reversibility criteria of dormancy [9]. MRD more broadly denotes residual tumor cells or tumor‐derived molecular signals that remain below the threshold for routine clinical detection [6]. These concepts overlap but should not be used interchangeably.

Across gynecologic malignancies, different tumor types exhibit distinct patterns of dissemination and recurrence, with only partially overlapping features related to dormancy‐associated biology. In high‐grade serous ovarian cancer (HGSOC), for example, transcoelomic spread commonly occurs through multicellular spheroids within the peritoneal cavity. These spheroids display slow‐cycling and therapy‐tolerant phenotypes, together with metabolic and extracellular matrix (ECM) adaptations, that may contribute to dissemination and recurrent disease; however, spheroid survival alone does not establish prolonged dormancy or reversible awakening [6, 10, 11]. In endometrial and cervical cancers, DTCs have also been identified in blood, bone marrow, and distant tissue sites, and some studies have linked their presence to a higher risk of distant recurrence or worse disease‐free survival [7, 8]. Nevertheless, DTC detection establishes the presence of disseminated residual disease but does not by itself demonstrate cellular quiescence, long‐term latency, or subsequent reversible reactivation. When residual tumor populations acquire low‐proliferative or dormancy‐like features, they may be less susceptible to conventional cytotoxic therapies that preferentially target rapidly dividing cells and may therefore contribute to delayed relapse [12, 13]. Direct gynecologic dormancy evidence is currently concentrated in ovarian cancer models, whereas evidence in endometrial and cervical cancers remains predominantly indirect.

Although tumor dormancy has been examined in several solid tumors, an integrated framework addressing the strength of available evidence, disease‐ and subtype‐specific heterogeneity, the validation of residual‐state biomarkers, and the translational use of spatial and longitudinal monitoring methods remains lacking for gynecologic malignancies. In this Review, we examine dormancy in gynecologic malignancies using entry, maintenance, and reactivation as an organizing framework rather than as a universally demonstrated or obligatory sequence. Across these phases, we evaluate candidate biological dependencies of dormancy‐associated residual disease, including redox regulation, lipid peroxidation‐buffering pathways, mitochondrial plasticity, adaptive stress‐response programs, epigenetic and post‐transcriptional regulation, immune interactions, and ECM sensing. Throughout the Review, we distinguish among direct gynecologic dormancy evidence, indirect gynecologic dormancy‐associated evidence, and cross‐cancer mechanistic extrapolation.

We therefore evaluate two biologically distinct investigational therapeutic concepts: suppression of reactivation signals and state modulation followed by biomarker‐defined treatment. These approaches have different objectives and risks and should not currently be regarded as interchangeable or inherently complementary strategies. We also discuss how spatial and single‐cell profiling can characterize local residual‐cell ecosystems, whereas longitudinal liquid biopsy can support MRD detection and longitudinal assessment of recurrence risk. Combining these approaches may support future inference of residual‐cell states and the development of biomarker‐guided clinical trials, but neither currently provides validated dormancy‐phase classification or independently determines treatment selection. Overall, this framework aims to connect residual‐cell biology with clinically testable hypotheses while maintaining clear distinctions between established evidence, candidate mechanisms, and future translational possibilities.

## 2 Deconstructing the Reversible Dynamics of Tumor Dormancy

Tumor dormancy should not be regarded as a single static event. Instead, it may involve dynamic and potentially reversible changes in cell‐cycle control, metabolism, and interactions with the host microenvironment. Available studies support the use of entry, maintenance, and awakening as an organizing model, although complete phase‐to‐phase trajectories have rarely been demonstrated in gynecologic tumors. This phase‐based framework provides a useful basis for understanding how residual disease persists after treatment and for identifying points at which translational interventions could be investigated.

### 2.1 Dormancy Entry: Stress‐Induced Defensive Reprogramming and Autophagy Dependence, and Mechanical Constraint

When tumor cells are exposed to chemotherapy, hypoxia, nutrient limitation, or matrix deprivation, a subset survives rather than undergoing immediate cell death and enters a stress‐adaptive state in which integrated stress response (ISR)‐ and unfolded protein response (UPR)‐related signaling functions as an upstream regulatory program [14, 15, 16]. This state is commonly associated with increased phosphorylation of eukaryotic translation initiation factor 2 alpha (eIF2α), global translational repression, selective translation of activating transcription factor 4 (ATF4), and induction of genes involved in antioxidant defense, metabolic adaptation, and autophagy‐related survival [14, 15, 16, 17]. These responses help tumor cells remain viable after loss of attachment and under nutrient restriction or therapeutic stress, which may support their transition into a dormancy‐like state [17]. However, these general stress‐survival programs do not by themselves establish dormancy entry in gynecologic malignancies.

Among gynecologic malignancies, ovarian cancer provides the most direct evidence linking dormancy entry to autophagy dependence. DIRAS family GTPase 3 (DIRAS3; also known as aplasia Ras homolog member I [ARHI]) has been identified as a regulator of this process. Re‐expression of DIRAS3 can induce a dormancy‐associated state in vivo and, through interaction with BECN1, promote formation of an autophagosome initiation complex containing PIK3C3, PIK3R4, and ATG14, thereby increasing autophagy and supporting survival of nutrient‐restricted, low‐proliferative residual cells [18, 19]. Three‐dimensional spheroid models relevant to peritoneal dissemination also provide functional evidence that autophagy contributes to survival under matrix deprivation. Chen et al. showed that BNIP3/BNIP3L‐associated autophagy promotes anoikis resistance in spheroid and patient‐derived spheroid models of ovarian cancer, whereas autophagy inhibition or disruption of BECN1 suppresses spheroid formation and survival [10]. These spheroid findings support persistence after matrix detachment but do not directly demonstrate prolonged dormancy, reversible awakening, or recurrence arising from a prospectively defined dormant population. Esposito et al. further showed in three‐dimensional ovarian cancer models that interleukin‐6 (IL‐6) suppresses ARHI/DIRAS3 through microRNA‐1305 (miR‐1305), impairs autophagy, and promotes spheroid outgrowth, whereas resveratrol reverses this effect and maintains a lower‐proliferative, more dormancy‐like state [20].

Mechanical constraint has also been examined in a direct ovarian cancer model of reversible dormancy‐associated behavior. Lam et al. showed that ovarian cancer cells immobilized within silica‐gel matrices acquired a low‐proliferative and chemoresistant phenotype, resumed proliferation after release, and were more likely to re‐enter a dormant state following subsequent immobilization [21]. Although this engineered system does not reproduce the full complexity of an in vivo niche, it supports the possibility that physical confinement can contribute to reversible dormancy entry.

Direct evidence for dormancy entry is more limited in other gynecologic tumors, but a similar stress‐adaptive pattern is emerging. In endometrial cancer, Ran et al. found that stem‐like spheres show high autophagic activity and that autophagy inhibition reduces stemness, self‐renewal, and paclitaxel tolerance, consistent with a stress‐tolerant survival state [22]. In cervical cancer, Meng et al. reported that radiation induces protective autophagy, and that autophagy inhibition increases radiation‐induced cytotoxicity and apoptosis while reducing formation of polyploid giant cancer cells, which have been associated with post‐treatment survival and recurrence potential [23]. These endometrial and cervical studies support therapy‐tolerant persistence but do not directly demonstrate long‐term dormancy entry or reversible awakening. Overall, the available evidence supports a working model in which ISR/UPR‐related reprogramming, autophagy, and mechanical constraint may contribute to dormancy entry or dormancy‐associated persistence, with direct evidence concentrated in ovarian cancer models.

### 2.2 Dormancy Maintenance: Metabolic Rewiring, Niche Support, and Immune Evasion

Once tumor cells enter a dormancy‐like or residual survival state, their primary requirement shifts from rapid proliferation to sustained survival despite low proliferative activity, limited nutrient availability, continued therapeutic pressure, and immune surveillance. Dormancy maintenance is therefore better understood as an actively maintained state characterized by metabolic rewiring, dependence on supportive local niches, and gradual acquisition of immune evasion [9]. Direct demonstration of maintenance‐defined by sustained low proliferation, continued viability, and the capacity for later reactivation‐ remains limited in gynecologic malignancies. MRD, drug‐tolerant persisters, spheroid survival, and chemoresistant populations are relevant to dormancy biology but should not be treated as equivalent to bona fide dormant cells.

In ovarian cancer, comparatively direct evidence of active dormancy maintenance is provided by inducible ARHI/DIRAS3 models. Lyu et al. compared ARHI‐induced dormant tumors with recurrent tumors arising after ARHI downregulation and identified coordinated changes in promoter methylation and histone modifications affecting angiogenesis‐ and adhesion‐related programs, including TIMP3 and CDH1 [24]. These findings indicate that ovarian tumor dormancy is associated with regulated and potentially reversible epigenetic states rather than solely with passive growth arrest, although this evidence remains preclinical.

Another recurring feature of post‐therapy residual cells, drug‐tolerant persisters, and MRD is a shift away from glycolysis toward increased reliance on fatty acid oxidation (FAO) and oxidative phosphorylation (OXPHOS). Artibani et al. showed that clinical MRD samples from high‐grade serous ovarian cancer display an adipocyte‐like gene signature enriched for lipid metabolism genes, indicating that lipid utilization is a defining feature of residual disease rather than a secondary metabolic change [25]. This phenotype has also been reproduced in functional models. Yang et al. established a three‐dimensional microtumor model of ovarian cancer MRD with transcriptional similarity to patient‐derived residual disease and demonstrated sensitivity to the FAO inhibitor perhexiline [26]. Lin et al. further reported that paclitaxel‐tolerant ovarian cancer persister cells show increased FAO, driven in part by CCAAT/enhancer‐binding protein beta (CEBPB)‐dependent hydroxyacyl‐CoA dehydrogenase trifunctional multienzyme complex subunit alpha (HADHA) expression, and that inhibition of this pathway restores paclitaxel sensitivity [27]. Nallanthighal et al. also showed that COL11A1 promotes fatty acid metabolism through the DDR2–Src–Akt–AMP‐activated protein kinase (AMPK) axis and supports cisplatin‐resistant survival, again indicating that FAO serves as a major metabolic support program in residual ovarian cancer cells [28]. Collectively, these studies identify a recurring dependency in MRD, persister, and chemoresistant ovarian cancer states, but do not establish that FAO is specifically required for long‐term dormancy maintenance.

Residual‐cell survival may also be supported by the microenvironment. In ovarian cancer, omental and adipose‐associated niches provide relevant examples. Lischetti et al. showed that even macroscopically uninvolved lesser omentum can display features of a premetastatic niche, including neutrophil infiltration, extracellular trap formation, and micrometastatic organization, indicating that the omentum is actively reprogrammed before overt tumor expansion [29]. Waters et al. further showed that omental preadipocytes induce insulin‐like growth factor‐binding protein 5 (IGFBP5) expression in ovarian cancer cells and enhance adhesion, clonogenicity, stress tolerance, and tumor‐initiating capacity through cAMP response element‐binding protein (CREB) signaling [30]. These findings support a role for niche‐derived signals in residual‐cell survival and metastatic competence but do not directly trace a dormant population through long‐term maintenance. In endometrial cancer, hypoxic and immunomodulatory niches may support related persistent states. Chen et al. showed that hypoxia maintains endometrial cancer stem‐like cells through hypoxia‐inducible factor (HIF)‐dependent induction of ALKBH5 and demethylation of SOX2 mRNA [31], whereas Yin et al. found that programmed death‐ligand 1 (PD‐L1) and programmed death‐ligand 2 (PD‐L2) are upregulated under hypoxia and contribute to sphere formation and stemness‐associated programs [32]. In addition, Wang et al. isolated endometrial cancer‐infiltrating mesenchymal stem cells and showed that they suppress peripheral blood mononuclear cell proliferation and cytotoxic phenotypes, supporting an immunosuppressive stromal contribution to residual disease maintenance [33]. These endometrial findings support persistence‐prone hypoxic, stem‐like, and immune‐suppressive states, but remain indirect evidence for dormancy maintenance. Overall, epigenetic regulation, flexible substrate use, niche support, and immune evasion are plausible contributors to long‐term persistence, although their phase‐specific causal roles require temporally resolved validation.

### 2.3 Dormancy Awakening: Systemic Stress Hormones, Inflammatory Signaling, and the Angiogenic Switch

Reactivation of dormant or residual tumor cells may contribute to late recurrence and remains difficult to study in clinical settings. The available evidence falls into three categories: direct ovarian‐inclusive models of dormant‐cell reactivation, indirect ovarian cancer studies identifying awakening‐permissive niches, and mechanistic hypotheses extrapolated from non‐gynecologic residual‐tumor models.

The most direct mechanistic evidence to date comes from studies of systemic stress signaling. Perego et al. showed in dormancy models that included ovarian cancer that stress hormones activate β‐adrenergic signaling in neutrophils, promoting release of S100A8/A9 and generation of myeloperoxidase (MPO)‐dependent oxidized lipids. These oxidized lipids then activate fibroblast growth factor receptor (FGFR) signaling in dormant tumor cells, thereby inducing their exit from dormancy and the formation of new tumor lesions. The same study also linked higher circulating S100A8/A9 levels to shorter time to recurrence and showed that inhibition of S100A8/A9 or β2‐adrenergic receptors suppresses stress‐induced reactivation [34]. These findings provide direct preclinical evidence for stress‐induced awakening in an ovarian‐inclusive setting, although the mechanism has not been validated across gynecologic cancer types or molecular subtypes. Lee et al. showed in orthotopic ovarian cancer models that neutrophil infiltration and neutrophil extracellular trap (NET) formation occur in the omentum before overt metastasis, thereby promoting tumor cell retention within a premetastatic niche [35]. The same group later showed that NETs induce CXCL13, expand innate‐like B cells, and promote interleukin‐10 (IL‐10)‐associated immunoregulatory signaling in the premetastatic omentum [36]. Related studies by Lischetti et al. identified neutrophil‐rich and extracellular trap‐associated reprogramming in human ovarian omental tissue, whereas Ghisoni et al. showed that recurrent ovarian cancer re‐establishes a myeloid‐dominant immune landscape [29, 37]. These ovarian studies identify inflammatory and immune conditions associated with colonization or recurrence, but they do not directly trace awakening of a prospectively defined dormant‐cell population.

Renewed angiogenic activity may also contribute to dormancy awakening. Cho et al. demonstrated that slow‐cycling chemoresistant cells activate the activating transcription factor 6–epidermal growth factor (ATF6–EGF) axis, which promotes endothelial migration, tube formation, and an angiogenic switch in residual tumors, thereby facilitating tumor regrowth [38]. In related work, the same group showed that these residual cells recruit fibroblasts, increase collagen deposition and prostaglandin E2 (PGE2) production, and establish a proinflammatory niche that supports further outgrowth [39]. Because these studies were performed in non‐gynecologic squamous‐cell carcinoma models, they should be regarded as cross‐cancer, hypothesis‐generating evidence rather than as evidence of validated mechanisms of gynecologic dormant‐cell awakening. These data support a working model in which late recurrence may involve coordinated changes in systemic physiology and local microenvironmental support rather than spontaneous regrowth of individual residual cells.

### 2.4 Disease‐ and Subtype‐Specific Evidence Landscape

Evidence supporting dormancy‐associated biology is unevenly distributed across gynecologic malignancies and should not be interpreted as uniformly applicable to different disease types or molecular subgroups. Ovarian cancer provides the largest body of direct preclinical dormancy evidence and indirect evidence from MRD, therapy‐tolerant populations, spheroid models, and recurrent‐tumor microenvironments [6, 25]. Within ovarian cancer, HGSOC is the most extensively studied translational context, but direct dormancy evidence in low‐grade serous, clear‐cell, endometrioid, and mucinous carcinomas remains comparatively sparse. Mechanisms identified in HGSOC should therefore not be assumed to apply across all ovarian histotypes [40, 41].

In endometrial cancer, evidence remains predominantly indirect and is derived from studies of hypoxia‐adapted stem‐like cells and immune‐checkpoint regulation [31, 32], immunosuppressive stromal niches [33], treatment resistance [42], and DTC studies [8]. Endometrial cancer is classified into DNA polymerase epsilon‐ultramutated, mismatch repair‐deficient/microsatellite‐instability‐high, no‐specific‐molecular‐profile/copy‐number‐low, and p53‐abnormal/copy‐number‐high groups, but differences among these groups in dormancy entry, maintenance, or reactivation have not been systematically defined [43].

Evidence in cervical cancer is likewise largely indirect and is concentrated in DTC detection, radiation‐tolerant survival, polyploid giant cancer‐cell formation, and treatment‐associated cellular or microenvironmental states [7, 23]. Cervical squamous‐cell carcinoma and adenocarcinoma display distinct stromal, endothelial, fibroblast, and macrophage architectures, and multi‐omic studies have identified histology‐ and treatment‐associated malignant‐cell ecosystems that have not been validated as dormancy phases [44, 45].

Gynecologic tumor dormancy should therefore be studied as a group of context‐dependent residual‐cell states rather than as a uniform pan‐gynecologic program. Prospective studies should stratify models and patients by cancer type, histology, molecular subtype, treatment exposure, and anatomical niche when defining dormancy‐associated biomarkers or therapeutic dependencies.

Entry, maintenance, and awakening provide an organizing framework for examining how residual gynecologic tumor cells survive treatment, persist over time, and may later resume growth. These phases should not be interpreted as a universally demonstrated or obligatory sequence, because the directness of evidence varies across gynecologic cancer types, histologic subtypes, and experimental contexts, with direct evidence concentrated in ovarian cancer models. Representative studies, classified according to evidence directness and disease context, are summarized in Table 1, while Figure 1 presents a conceptual overview of this phase‐based framework.

**TABLE 1:** Evidence classification of studies relevant to dormancy entry, maintenance, and awakening in gynecologic malignancies.

Phase	Cancer type	Evidence class	Process; representative study and model	Main finding	Interpretation/limitation
Entry	Ovarian cancer	Direct	DIRAS3‐associated autophagy; Lu et al. [18, 19], inducible ovarian cell and in vivo models	DIRAS3 induces a dormancy‐associated state and BECN1‐containing autophagosome initiation.	Direct but model‐dependent preclinical evidence; clinical utility of autophagy‐directed intervention is unvalidated.
Entry	Ovarian cancer	Indirect	Anoikis resistance; Chen et al. [10], 3D and patient‐derived spheroids	BNIP3/BNIP3L‐associated autophagy supports spheroid survival during matrix deprivation.	Supports detached‐cell persistence, not prolonged dormancy or reversible awakening.
Entry	Ovarian cancer	Direct	Reversible DIRAS3 state control; Esposito et al. [20], 3D ovarian model	IL‐6/miR‐1305 suppresses DIRAS3 and promotes outgrowth; resveratrol reverses this response.	Supports a reversible dormancy‐associated transition in vitro; clinical relevance remains unvalidated.
Entry	Ovarian cancer	Direct	Mechanical confinement; Lam et al. [21], silica‐matrix immobilization	Immobilized cells become low‐proliferative and chemoresistant, resume growth after release, and can re‐enter dormancy.	Demonstrates reversibility in an engineered system that does not reproduce the native niche.
Entry	Endometrial cancer	Indirect	Stress‐adaptive autophagy; Ran et al. [22], stem‐like spheres	Autophagy supports stemness, self‐renewal, and paclitaxel tolerance.	Models therapy‐tolerant persistence rather than long‐term dormancy entry.
Entry	Cervical cancer	Indirect	Radiation‐associated protective autophagy; Meng et al. [23], treated cervical cells	Autophagy inhibition increases apoptosis and reduces polyploid giant cancer‐cell formation.	Supports post‐treatment persistence, not prospectively defined dormancy or awakening.
Maintenance	Ovarian cancer	Direct	Reversible epigenetic regulation; Lyu et al. [24], inducible dormant/recurrent tumors	Dormant and recurrent tumors show state‐dependent promoter methylation and histone changes affecting angiogenesis and adhesion.	Direct but preclinical evidence for active epigenetic maintenance and recurrence‐associated state change.
Maintenance	Ovarian cancer	Indirect	FAO/OXPHOS in MRD and persisters; Artibani, Yang, and Lin et al. [25, 26, 27]	Clinical MRD and experimental residual/persister models show lipid‐metabolic adaptation and functional FAO dependence.	Defines candidate residual‐state metabolism; long‐term dormancy specificity is unproven.
Maintenance	Ovarian cancer	Indirect	Omental/adipose niche support; Lischetti and Waters et al. [29, 30]	Omental reprogramming and preadipocyte‐derived signals enhance adhesion, stress tolerance, and tumor initiation.	Supports persistence‐prone niche biology without longitudinal tracking of dormant cells.
Maintenance	Endometrial cancer	Indirect	Hypoxia, stemness, and stromal immune suppression; Chen, Yin, and Wang et al. [31, 32, 33]	Hypoxia sustains SOX2 and PD‐L1/PD‐L2 programs; cancer‐associated stromal cells suppress immune effectors.	Supports persistence‐prone states; direct dormancy maintenance remains unestablished.
Awakening	Ovarian‐inclusive model	Direct	Stress hormone‐neutrophil signaling; Perego et al. [34], ovarian‐inclusive dormancy models	β‐adrenergic signaling induces neutrophil‐derived oxidized lipids and FGFR‐dependent awakening.	Direct preclinical awakening evidence, but not validated across gynecologic diseases or subtypes.
Awakening	Ovarian cancer	Indirect	NET‐associated premetastatic and immunoregulatory niche; Lee et al. [35, 36]	NETs precede visible metastasis, facilitate omental colonization, and induce CXCL13/IL‐10‐associated immune remodeling.	Defines recurrence‐permissive niche remodeling without tracing a prospectively defined dormant population.
Awakening	Ovarian cancer	Indirect	Human recurrence‐associated immune remodeling; Lischetti and Ghisoni et al. [29, 37]	Myeloid‐rich and trap‐associated remodeling is observed before or during metastatic recurrence.	Cross‐sectional or recurrence‐associated evidence; phase‐resolved causality remains limited.
Awakening	Non‐gynecologic model	Cross‐cancer	Angiogenic/fibroinflammatory switch; Cho et al. [38, 39], residual SCC models	ATF6‐EGF signaling, angiogenesis, fibroblast recruitment, collagen deposition, and PGE2 support regrowth.	Hypothesis‐generating mechanism requiring validation in gynecologic dormant‐cell models.

Abbreviations: 3D, three‐dimensional; FAO, fatty acid oxidation; FGFR, fibroblast growth factor receptor; HGSOC, high‐grade serous ovarian cancer; MRD, minimal residual disease; NET, neutrophil extracellular trap; OXPHOS, oxidative phosphorylation; PGE2, prostaglandin E2; SCC, squamous‐cell carcinoma.

**FIGURE 1:**
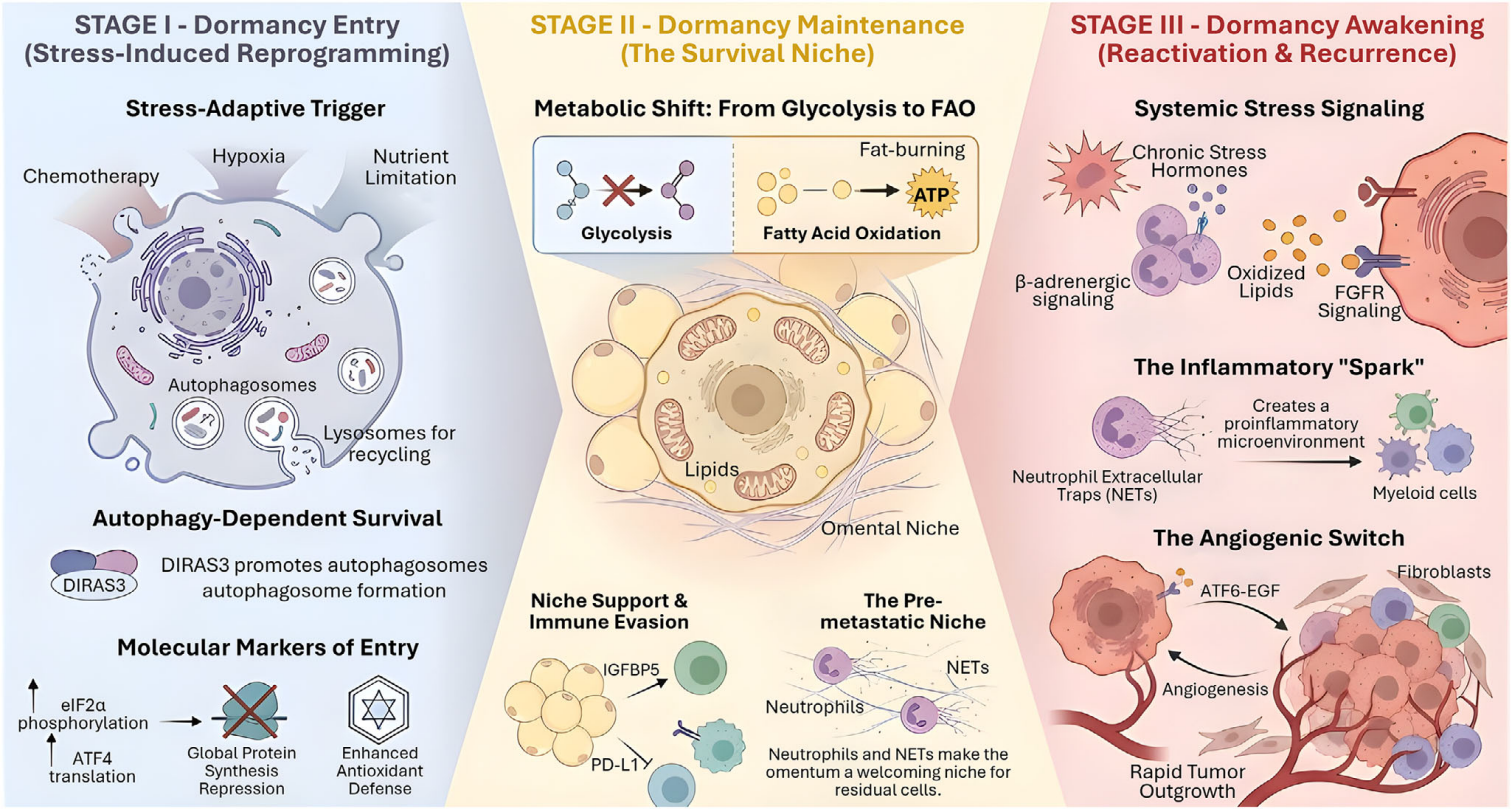
The reversible lifecycle of tumor dormancy in gynecologic cancers. The progression of tumor dormancy is characterized by three distinct functional stages: (Stage I) Dormancy Entry, where stress‐adaptive triggers (chemotherapy, hypoxia, and nutrient limitation) induce metabolic and molecular reprogramming. This phase is driven by DIRAS3‐mediated autophagy and molecular markers such as eIF2α phosphorylation and ATF4 translation, leading to global protein synthesis repression and enhanced antioxidant defense. (Stage II) Dormancy maintenance occurs within the survival niche (the omentum), involving a metabolic shift from glycolysis to fatty acid oxidation (FAO). The omental niche is further reprogrammed by neutrophils and neutrophil extracellular traps (NETs) to facilitate immune evasion (via PD‐L1) and niche support. (Stage III) Dormancy awakening is triggered by systemic stress signaling and an inflammatory “Spark.” Chronic stress hormones and β‐adrenergic signaling converge to activate the angiogenic switch, involving the ATF6‐EGF axis and fibroblast recruitment, ultimately resulting in rapid tumor outgrowth and clinical recurrence.

## 3 Candidate Biological Dependencies in Dormancy‐Associated Residual Disease

Based on recent primary studies, the candidate biological dependencies of dormancy‐associated residual tumor cells can be grouped into seven interrelated biological modules: redox control and nuclear factor erythroid 2‐related factor 2 (NRF2)‐linked metabolic dependency, lipid peroxidation buffering and ferroptosis resistance, mitochondrial plasticity and bioenergetic switching, adaptive stress‐response programs and proteostasis, epigenetic and post‐transcriptional plasticity, immune‐mediated equilibrium and escape, and extracellular‐matrix remodeling and mechanobiology. These features should not be regarded as isolated categories. Instead, they form a functional network that supports the survival, treatment tolerance, and reactivation potential of residual cells. Most available gynecologic studies examine MRD, persister, chemoresistant, spheroid, or recurrence‐associated states rather than longitudinally validated dormant‐cell populations; the following pathways are therefore presented as candidate dependencies rather than established actionable vulnerabilities.

### 3.1 Redox Control and Antioxidant Adaptation

To remain viable under low‐proliferative conditions, dormancy‐associated residual tumor cells may depend on maintenance of redox homeostasis in the setting of persistent oxidative stress and therapy‐induced damage. In dormant and recurrent tumor models, Fox et al. showed that NRF2 activation promotes redox balance and de novo nucleotide synthesis, while rendering recurrent cells selectively sensitive to glutaminase inhibition, indicating that NRF2‐driven redox adaptation may represent a targetable metabolic dependency [46]. This direct dormancy mechanism was established outside gynecologic cancer and should be treated as cross‐cancer mechanistic evidence.

In gynecologic malignancies, although direct mechanistic studies focused on dormancy maintenance remain limited, primary evidence supports a link between NRF2 pathway activation and treatment resistance [47]. Konstantinopoulos et al. reported that Kelch‐like ECH‐associated protein 1 (KEAP1)/NRF2 pathway activation is associated with carboplatin resistance in ovarian cancer [48], whereas Huang et al. showed that miR‐181d activates NRF2/antioxidant response element (ARE) signaling through KEAP1 degradation and thereby promotes cisplatin resistance [49]. In MYC‐high HGSOC, Raninga et al. further showed that a highly redox‐buffered state can be accompanied by compensatory glutamine metabolism that is suppressible with the glutaminase 1 (GLS1) inhibitor CB‐839 [50]. In line with this, Šimčíková et al. found that primary HGSOC cells show marked metabolic plasticity under glucose‐ or glutamine‐limited conditions, supporting the biological plausibility of glutamine dependency in stress‐adapted residual states [51]. In endometrial cancer, Jiang et al. likewise linked higher NRF2 expression to chemoresistance in type II tumors [42].

Taken together, these findings identify NRF2‐centered antioxidant signaling and glutamine‐associated metabolism as candidate dependencies of treatment‐tolerant and residual gynecologic cancer states, but not yet as validated requirements of long‐term dormant MRD.

### 3.2 Lipid Peroxidation‐Buffering Pathways and Ferroptosis Resistance

Residual and therapy‐tolerant tumor cells may depend on limiting lipid peroxidation to avoid ferroptotic cell death. Primary studies indicate that resistant or persister‐like ovarian cancer cells often show increased lipid metabolic regulation and ferroptosis‐buffering capacity, which allows survival under oxidative and therapeutic stress. These studies primarily model resistant, persister‐like, ascites‐derived, or genetically defined states rather than bona fide dormant cells.

In ovarian cancer, Tesfay et al. demonstrated that the lipid desaturase stearoyl‐CoA desaturase 1 (SCD1) increases the proportion of membrane monounsaturated fatty acids, reduces lipid peroxide accumulation, and protects ovarian cancer cells from RSL3‐ or erastin‐induced ferroptosis [52]. Xuan et al. further showed in ascites‐derived ovarian cancer cells that fatty acid desaturation mediated by SCD1 and fatty acid desaturase 2 (FADS2) maintains lipid metabolic balance and redox homeostasis, whereas inhibition of these enzymes lowers glutathione peroxidase 4 (GPX4) levels, decreases the glutathione/glutathione disulfide (GSH/GSSG) ratio, increases lipid peroxidation, and induces redox‐driven ferroptosis [53]. Luo et al. identified PAX8 as another regulator of ferroptosis resistance, showing that PAX8 depletion sensitizes ovarian cancer cells to GPX4 inhibition through suppression of glutamate‐cysteine ligase catalytic subunit (GCLC) and impaired glutathione synthesis [54]. Wang et al. reported that frizzled class receptor 7‐positive platinum‐tolerant ovarian cancer cells show altered glutathione metabolism and GPX4 dependence, rendering them selectively sensitive to ferroptosis induction [55]. More recently, Xie et al. demonstrated in BRCA1‐deficient ovarian cancer that poly (ADP‐ribose) polymerase (PARP) inhibitors can themselves induce ferroptosis, and that inhibition of GPX4‐mediated protection further increases lipid peroxidation and cell death in patient‐derived organoids and xenografts [56].

Although the strongest evidence at present comes from ovarian cancer, Fan et al. reported that downregulation of TSPAN1 promotes ferroptosis in endometrial cancer cells and reduces malignant phenotypes, suggesting that ferroptosis‐buffering pathways may also contribute to survival in other gynecologic tumors [57]. Taken together, SCD1/FADS2‐mediated desaturation, PAX8–GCLC–glutathione signaling, and GPX4‐dependent control are candidate dependencies of ferroptosis‐resistant and treatment‐tolerant gynecologic cancer states. Direct evidence that ferroptosis induction selectively eliminates long‐term dormant gynecologic MRD remains limited.

### 3.3 Mitochondrial Plasticity and Bioenergetic Switching

The ability of dormancy‐like, residual, or therapy‐resistant tumor cells to survive hypoxia, nutrient deprivation, and continued treatment pressure is closely linked to mitochondrial plasticity and flexible substrate use. Their survival advantage reflects not only reduced proliferation, but also the ability to switch among glucose, fatty acids, and mitochondrial respiration to maintain adenosine triphosphate (ATP) production, limit oxidative damage, and preserve treatment tolerance. Most gynecologic evidence, however, derives from chemoresistant cells, detached spheroids, or drug‐tolerant persister models rather than temporally validated dormant‐cell populations.

In ovarian cancer, Tan et al. showed that platinum‐resistant cells have increased uptake of exogenous fatty acids and partial dependence on β‐oxidation, and that inhibition of carnitine palmitoyltransferase 1 (CPT1)/FAO can restore platinum sensitivity [58]. Nallanthighal et al. further demonstrated that COL11A1 drives fatty acid β‐oxidation and promotes cisplatin resistance, which can be reversed by FAO inhibition [28]. In HGSOC models, Sawyer et al. found that attachment loss and suspension growth induce CPT1A expression and FAO dependence, thereby supporting anoikis resistance and spheroid survival [11]. Lin et al. extended this observation by showing that paclitaxel‐tolerant ovarian cancer persister cells exhibit elevated FAO and become resensitized to paclitaxel following HADHA suppression or pharmacologic FAO blockade [27]. Dar et al. also reported that chemoresistant ovarian cancer cells have higher OXPHOS capacity, with palmitate‐induced oxygen consumption suppressed by etomoxir, consistent with dependence on fatty acid‐driven mitochondrial respiration [59].

Mitochondrial dynamics also contribute to treatment resistance. Han et al. showed that hypoxia promotes reactive oxygen species (ROS)‐dependent mitochondrial fission and cisplatin resistance in ovarian cancer cells, whereas dynamin‐related protein 1 (DRP1) inhibition restores drug sensitivity under hypoxic conditions [60]. Similarly, Zou et al. found that altered DRP1 and mitofusin 2 (MFN2) expression reshapes mitochondrial dynamics in SKOV3/DDP cells and contributes to stable cisplatin resistance [61]. Taken together, these studies establish FAO, OXPHOS, and mitochondrial dynamics as determinants of survival in selected ovarian spheroid, persister, and chemoresistant states, but not as universal features or validated therapeutic targets of dormant gynecologic tumor cells.

### 3.4 Adaptive Stress Responses and Proteostasis

The UPR is a major adaptive mechanism that allows tumor cells to maintain proteostasis under hypoxia, nutrient deprivation, and treatment‐induced stress. In gynecologic malignancies, primary studies indicate that the UPR is not merely a bystander response, but a functionally important node in tumor survival, protective autophagy, and chemoresistance. Most of these studies examine proliferative, chemoresistant, or stress‐treated populations rather than bona fide dormant cells.

In HGSOC, Meng et al. showed that the ID1‐IL‐6/STAT3‐ATF6 axis induces protective autophagy and increases resistance to cisplatin and paclitaxel. They also found that higher ATF6 expression in a cohort of 1104 HGSOC cases was associated with platinum resistance and shorter overall and progression‐free survival (PFS) [62]. Samanta et al. further reported that glucose‐regulated protein 78 (GRP78), protein kinase R–like ER kinase (PERK), and ATF6 are markedly upregulated in serous and clear cell ovarian carcinoma relative to normal tissue, and that high expression of GRP78 and protein disulfide isomerase was associated with worse outcome [63].

In endometrial cancer cells, Conza et al. found that metformin disrupts pro‐survival UPR signaling by downregulating GRP78 and activating the ATF4/C/EBP homologous protein (CHOP) axis, thereby suppressing cell growth and promoting apoptosis [64]. In cervical cancer, Taguchi et al. showed that Yip1A sustains inositol‐requiring enzyme 1 (IRE1) and PERK signaling, whereas inhibition of Yip1A induces apoptosis and reduces anti‐apoptotic and autophagy‐related responses [65]. Collectively, these findings establish UPR and proteostasis pathways as mediators of treatment tolerance and stress adaptation, but do not directly demonstrate durable dormancy or phase‐specific dependence. They should therefore be considered candidate dependencies of stress‐adapted residual disease.

### 3.5 Epigenetic and Post‐Transcriptional Plasticity

Because dormancy is potentially reversible, its establishment, maintenance, and reactivation are likely to involve dynamic regulation of DNA methylation, histone modification, chromatin accessibility, transcriptional memory, RNA stability, and translation. In ovarian cancer, Lyu et al. showed that ARHI/DIRAS3‐induced dormant tumors and recurrent tumors arising after ARHI downregulation display state‐dependent promoter methylation and histone modifications affecting angiogenesis‐ and adhesion‐related genes [24]. This provides direct but model‐dependent evidence that ovarian dormancy is accompanied by reversible epigenetic remodeling.

Cross‐cancer models further implicate NR2F1‐associated quiescence, macroH2A‐mediated suppression of metastatic outgrowth, oncogenic enhancer‐mediated transcriptional memory, and NR2F1‐AS1/PTBP1‐dependent post‐transcriptional control in dormant‐cell regulation [66, 67, 68, 69]. In gynecologic cancers, IL‐6/miR‐1305‐mediated suppression of DIRAS3 promotes ovarian spheroid outgrowth [20], whereas ALKBH5‐associated regulation of SOX2 supports a hypoxia‐adapted stem‐like state in endometrial cancer [31]. These findings implicate non‐coding RNA and RNA modification in residual‐state plasticity, but only the ovarian DIRAS3 study directly addresses a reversible dormancy‐associated transition. Matched single‐cell transcriptomic and chromatin‐accessibility profiling further demonstrates substantial regulatory heterogeneity across ovarian and endometrial cancers [70], providing an enabling framework for identifying candidate dormancy‐associated regulatory states. However, no gynecologic dormancy‐specific epigenetic or post‐transcriptional signature has yet been prospectively validated. Epigenetic and RNA‐regulatory pathways should therefore be considered candidate state‐control mechanisms requiring temporally resolved validation.

### 3.6 Immune‐Mediated Dormancy, Equilibrium, and Escape

Dormancy‐like, residual, and recurrence‐prone tumor cells do not evade immune surveillance only through passive escape. Rather, they can remodel the tumor microenvironment through metabolic and lipid oxidation‐related signals, gradually establishing an immunosuppressive state that favors long‐term persistence. Primary studies indicate that oxidized lipids and lipid peroxidation products act not only as by‐products of oxidative stress, but also as immunomodulatory signals that affect dendritic cells (DCs), myeloid‐derived suppressor cells (MDSCs), and effector T cells [71].

In ovarian cancer, Cubillos‐Ruiz et al. demonstrated that sustained XBP1 activation in tumor‐associated DCs drives abnormal lipid metabolism and impairs antigen presentation, thereby suppressing antitumor T‐cell immunity. The lipid peroxidation product 4‐hydroxynonenal (4‐HNE) was also sufficient to induce endoplasmic reticulum (ER) stress responses in DCs, linking lipid oxidation directly to defective antigen presentation [71]. Ugolini et al. further showed that polymorphonuclear MDSCs transfer oxidized truncated lipids to DCs and selectively suppress cross‐presentation in an MPO‐dependent manner, whereas MPO inhibition restores cross‐presentation and improves the effect of checkpoint blockade [72].

In a more direct dormancy‐reactivation context, Perego et al. demonstrated that stress‐activated neutrophils release S100A8/A9 and generate MPO‐dependent oxidized lipids that drive dormant lung and ovarian tumor cells out of dormancy [34]. Oxidized lipids also impair adaptive immunity. Xu et al. reported that intratumoral CD8^+^ T cells acquire oxidized lipids through CD36, leading to accumulation of lipid peroxidation, functional exhaustion, and reduced antitumor activity [73]. Hsu et al. further showed that 4‐HNE suppresses NLRP3 inflammasome activation and pyroptosis in macrophages, supporting a broader immunomodulatory role for lipid peroxidation products [74].

Additional evidence distinguishes immune‐mediated equilibrium from immune escape. Cross‐cancer studies show that tissue‐resident memory CD8^+^ T cells (TRM) can maintain prolonged tumor–immune equilibrium and restrain the outgrowth of occult tumor cells [75]. Macrophages can also regulate dormancy in a context‐dependent manner: primary‐tumor‐associated macrophages can prime disseminating tumor cells for a pro‐dormancy phenotype, whereas lung‐resident alveolar macrophages can impose dormancy at the metastatic site; subsequent loss of tumor‐cell responsiveness to these macrophage‐derived signals may permit metastatic outgrowth [76, 77]. In HGSOC, TRM states and omental macrophage subsets provide disease‐relevant evidence for immune control and metastatic niche support, respectively [78, 79]. These gynecologic findings characterize immune ecosystems associated with persistence or colonization but do not yet longitudinally demonstrate immune maintenance of dormant MRD.

Collectively, immune‐mediated dormancy in gynecologic malignancies remains a biologically plausible but incompletely validated framework. Temporally resolved studies are required to distinguish immune states that actively maintain equilibrium from those that permit immune escape, metastatic colonization, or reactivation.

### 3.7 Extracellular Matrix Remodeling and Mechanobiology

The ECM is an active regulator of residual‐cell behavior through its composition, architecture, stiffness, and mechanical signaling. Matrix cues influence integrin engagement, focal‐adhesion signaling, cytoskeletal tension, yes‐associated protein / transcriptional co‐activator with PDZ‐binding motif (YAP/TAZ) activity, and stromal or vascular interactions, but their direct roles in gynecologic tumor dormancy remain incompletely defined.

Direct ovarian evidence is provided by the mechanically constrained model of Lam et al., in which silica‐matrix immobilization selected a low‐proliferative, chemoresistant population capable of resuming growth after release and re‐entering dormancy following re‐immobilization [21]. Cross‐cancer studies further show that type I collagen can promote awakening through β1‐integrin/SRC/focal adhesion kinase (FAK)/extracellular signal‐regulated kinase (ERK), whereas a type III collagen‐rich matrix can support dormancy through discoidin domain receptor 1 (DDR1)‐associated signaling [80, 81]. These opposing effects emphasize that matrix identity and receptor context are as important as matrix abundance or stiffness.

In ovarian cancer, increased matrix rigidity and specific ECM substrates promote spheroid disaggregation, platinum resistance, and YAP‐associated mechanotransduction through Rho‐associated coiled‐coil‐containing kinase (ROCK), β1‐integrin, FAK, phosphorylated myosin light chain (pMLC), and YAP signaling [82, 83]. These studies support adhesion‐adapted and chemoresistant states rather than bona fide dormancy transitions. Omental tissue reprogramming and preadipocyte‐derived support may enhance adhesion, stress tolerance, and tumor‐initiating capacity [29, 30], whereas NET‐associated and myeloid immune remodeling may facilitate premetastatic colonization or recurrence‐associated outgrowth [35, 36, 37]. However, these studies do not provide phase‐resolved causal evidence of dormancy maintenance or reactivation.

ECM remodeling and mechanotransduction should therefore be considered context‐dependent candidate niche dependencies. Their use as dormancy‐specific therapeutic targets will require longitudinal models that distinguish maintenance of viable dormant cells from general suppression of growth, implantation, or metastatic colonization.

Taken together, these seven interlinked modules provide a framework for how residual gynecologic cancer cells survive therapy, tolerate prolonged stress, and retain reactivation potential. However, evidence directness varies: limited direct ovarian dormancy evidence supports epigenetic regulation and mechanical confinement, whereas most evidence for redox, ferroptosis, mitochondrial, UPR, immune, and ECM pathways comes from resistant, persister, spheroid, residual‐disease, or cross‐cancer models. These pathways should therefore be viewed as candidate biological dependencies rather than validated dormancy‐specific therapeutic vulnerabilities. Table 2 summarizes the evidence, mechanisms, investigational directions, and limitations, while Figure 2 illustrates their context‐dependent crosstalk.

**TABLE 2:** Candidate biological dependencies in dormancy‐associated residual disease: evidence base, investigational directions, and limitations.

Biological module	Evidence base	Representative mechanisms and biological role	Investigational direction	Principal limitation
Redox control and antioxidant adaptation	Direct cross‐cancer dormancy evidence [46]; indirect ovarian/endometrial resistance evidence [42, 47, 48, 49, 50, 51]	NRF2/KEAP1‐GLS1 and glutamine metabolism maintain redox balance, nucleotide supply, and treatment tolerance.	Test redox‐ or glutamine‐targeted combinations in biomarker‐defined residual‐state models.	No validated gynecologic dormancy‐specific dependency or patient‐selection biomarker.
Lipid‐peroxidation buffering and ferroptosis resistance	Indirect ovarian and emerging endometrial resistant‐state evidence [52, 53, 54, 55, 56, 57]	SCD1/FADS2, GPX4, GCLC/GSH, and PAX8 limit lipid peroxidation and ferroptotic death.	Evaluate ferroptosis sensitization, including PARPi combinations, in defined genetic and residual‐state contexts.	Evidence mainly derives from resistant, ascites‐derived, or genetically selected models rather than dormant MRD.
Mitochondrial plasticity and bioenergetic switching	Indirect ovarian spheroid, persister, and chemoresistance evidence [11, 27, 28, 58, 59, 60, 61]	CPT1A/HADHA‐dependent FAO, OXPHOS, and DRP1/MFN2 dynamics support ATP production, substrate flexibility, and therapy tolerance.	Test metabolic or mitochondrial‐dynamics modulation with state‐specific functional readouts.	Longitudinal roles across entry, maintenance, and reactivation remain undefined.
Adaptive stress responses and proteostasis	Indirect ovarian, endometrial, and cervical stress/resistance evidence [62, 63, 64, 65]	ATF6, GRP78, PERK, IRE1, ID1‐IL6/STAT3, and autophagy preserve proteostasis under treatment and hypoxic stress.	Evaluate UPR or autophagy modulation in residual‐disease models with viability and reactivation endpoints.	Most studies examine proliferative or therapy‐resistant cells, not bona fide dormant populations.
Epigenetic and post‐transcriptional plasticity	Direct ovarian model evidence [24]; cross‐cancer dormancy evidence [66, 67, 68, 69]; indirect gynecologic evidence [20, 31, 70]	DNA methylation, histone modification, macroH2A, NR2F1, enhancer memory, and RNA regulation enable reversible state transitions.	Develop temporally resolved chromatin/RNA signatures; test state modulation only with defined clearance strategies.	No prospectively validated gynecologic dormancy‐specific regulatory signature.
Immune‐mediated equilibrium and escape	Ovarian‐inclusive reactivation evidence [34]; cross‐cancer equilibrium/dormancy evidence [75, 76, 77]; indirect ovarian ecosystem evidence [71, 72, 73, 74, 78, 79]	TRM CD8^+^ T cells, NK cells, macrophages, XBP1, MPO, oxidized lipids, and CD36 may maintain equilibrium or permit escape.	Functionally distinguish equilibrium‐maintaining from escape‐permissive immune states before testing niche‐directed therapy.	Gynecologic studies rarely track dormant MRD longitudinally through immune maintenance and escape.
ECM remodeling and mechanobiology	Direct ovarian confinement evidence [21]; cross‐cancer dormancy/awakening evidence [80, 81]; indirect ovarian evidence [29, 30, 35, 36, 37, 82, 83]	Collagen I/III, DDR1, beta1‐integrin‐FAK, ROCK/pMLC, and YAP/TAZ regulate restraint, adhesion, persistence, or outgrowth.	Test matrix‐ and receptor‐specific interventions in longitudinal models that distinguish dormancy from general growth inhibition.	Matrix effects are context‐dependent; most ovarian evidence concerns adhesion, colonization, or chemoresistance.

*Note*: Candidate intervention directions are hypothesis‐generating and should not be interpreted as established dormancy‐specific therapeutic vulnerabilities.

Abbreviations: ECM, extracellular matrix; FAO, fatty acid oxidation; FAK, focal adhesion kinase; GSH, glutathione; MRD, minimal residual disease; OXPHOS, oxidative phosphorylation; PARPi, poly (ADP‐ribose) polymerase inhibitor; ROS, reactive oxygen species; TRM, tissue‐resident memory T cells; UPR, unfolded protein response.

**FIGURE 2:**
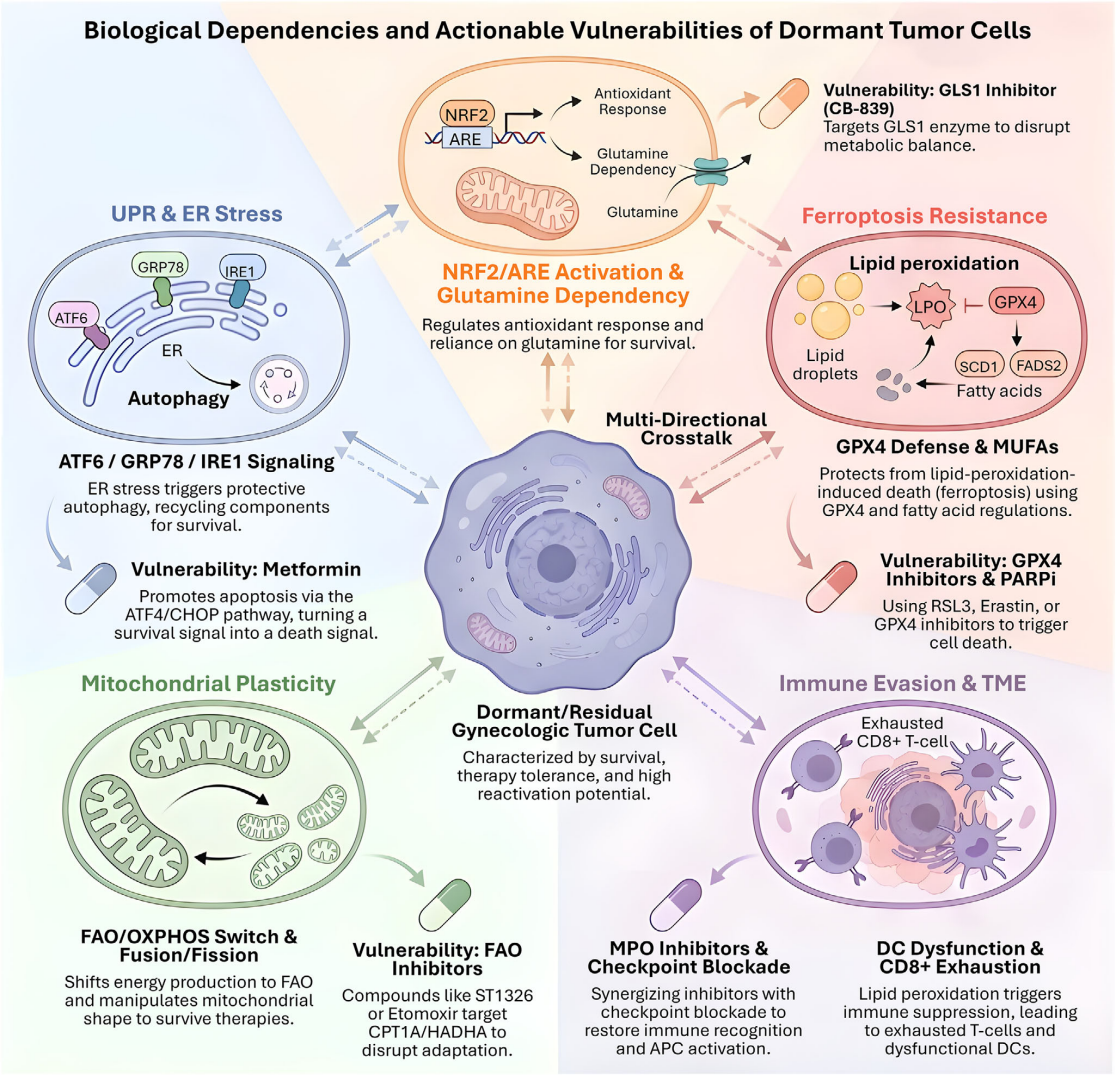
Candidate biological dependencies and context‐dependent crosstalk in dormancy‐associated residual disease. Dormancy‐associated residual gynecologic tumor cells may persist via interconnected programs involving: (1) adaptive stress responses and proteostasis; (2) redox control and antioxidant adaptation; (3) lipid‐peroxidation buffering and ferroptosis resistance; (4) mitochondrial plasticity and bioenergetic switching; (5) ECM remodeling and mechanobiology; (6) epigenetic and post‐transcriptional plasticity; and (7) immune‐mediated equilibrium and escape. Bidirectional dashed arrows indicate potential, context‐dependent crosstalk rather than universal co‐dependence. These pathways represent candidate biological dependencies rather than validated dormancy‐specific therapeutic vulnerabilities. Abbreviations: PERK, protein kinase R–like ER kinase; IRE1, inositol‐requiring enzyme 1; ATF6, activating transcription factor 6; GRP78 (BiP), glucose‐regulated protein 78 (binding immunoglobulin protein); ER, endoplasmic reticulum; NRF2, nuclear factor erythroid 2–related factor 2; ARE, antioxidant response element; GLS1, glutaminase 1; GPX4, glutathione peroxidase 4; SCD1, stearoyl‐CoA desaturase 1; FADS2, fatty acid desaturase 2; GCLC, glutamate–cysteine ligase catalytic subunit; GSH, glutathione; FAO, fatty acid oxidation; OXPHOS, oxidative phosphorylation; CPT1A, carnitine palmitoyltransferase 1 A; HADHA, hydroxyacyl‐CoA dehydrogenase/3‐ketoacyl‐CoA thiolase/enoyl‐CoA hydratase (trifunctional) α subunit; MFN2, mitofusin 2; DRP1, dynamin‐related protein 1; ECM, extracellular matrix; DDR1, discoidin domain receptor 1; FAK, focal adhesion kinase; YAP/TAZ, yes‐associated protein/transcriptional co‐activator with PDZ‐binding motif; TRM, tissue‐resident memory T cells.

## 4 Therapeutic Concepts for Dormancy‐Associated Residual Disease: Evidence, Risks, and Decision Constraints

Mechanistic studies of tumor dormancy and residual‐cell persistence have generated potential concepts for recurrence interception, but they do not yet support validated phase‐specific treatment assignment in gynecologic malignancies. Suppression of reactivation signals and state modulation followed by biomarker‐defined treatment have distinct biological objectives and risks and should therefore be regarded as separate investigational concepts rather than interchangeable or inherently complementary strategies.

Current management remains guided by cancer type, stage, histology, molecular subtype, treatment history, and response, and includes surgery, platinum‐based chemotherapy, radiotherapy, PARP inhibitors (PARPi), anti‐angiogenic therapy, immunotherapy, hormonal therapy, and disease‐specific maintenance treatment [84, 85, 86, 87]. Dormancy‐directed interventions should be evaluated as potential extensions of these established pathways rather than substitutes for standard care. Conventional maintenance therapy should not be equated with deliberate biological maintenance of tumor dormancy, because current regimens are not selected using validated dormant‐cell biomarkers.

### 4.1 Investigational Suppression of Awakening Signals to Prolong Clinical Latency

The aim of this strategy is to interrupt the intrinsic and extrinsic cues that drive dormant or residual tumor cells back into proliferative growth, thereby delaying or possibly preventing clinical recurrence. Based on the primary studies reviewed above, the most relevant translational entry points at present include systemic stress‐associated signaling, metabolic dependencies linked to reactivation, and angiogenic switching in residual tumors. This concept should be interpreted as suppression of reactivation‐associated signals rather than as validated maintenance of a clinically identifiable dormant‐cell state.

First, prior studies indicate that stress‐related β2‐adrenergic‐neutrophil signaling can promote reactivation of dormant tumor cells. This suggests that β‐adrenergic blockade or intervention against S100A8/A9‐associated inflammatory signaling may represent candidate approaches for prolonging clinical latency. In gynecologic cancer, however, these approaches remain mechanism‐based translational concepts, and current evidence is insufficient to support their use as standard maintenance therapy [34]. Second, dormancy and recurrence models have shown that a subset of residual cells may acquire NRF2‐related redox adaptation together with glutamine‐linked metabolic dependency during reactivation. On this basis, glutaminase inhibition may represent another possible strategy for dormancy interception. However, the most direct mechanistic evidence currently comes mainly from breast cancer models. In gynecologic malignancies, this approach is therefore better regarded as a biologically plausible candidate strategy rather than one established directly in disease‐specific dormancy models [46]. Third, reactivation of residual tumors may also depend on renewed angiogenic support. Cho et al. showed that slow‐cycling chemoresistant cells can activate endothelial cells through an ATF6‐EGF axis and promote an angiogenic switch in residual tumors, thereby facilitating tumor regrowth and recurrence [38, 39]. Although these data were generated in a non‐gynecologic residual tumor model, the underlying concept—that recurrence may be delayed by disrupting the supportive microenvironment required for reactivation—remains relevant to MRD‐directed strategies in gynecologic malignancies. In addition, recent ovarian cancer studies support the presence of local microenvironmental remodeling before overt recurrence [29, 35, 36, 37], implying that latency‐prolonging strategies should not focus only on residual tumor cells themselves, but also on the formation and maintenance of a niche permissive for awakening.

Taken together, the purpose of suppressing awakening signals is not to eliminate all residual cells immediately, but to reduce the likelihood that they re‐enter active proliferation. At present, this approach is best viewed as a mechanism‐based strategy for recurrence interception rather than an established component of routine clinical management. Long‐term suppression may require prolonged treatment and may preserve rather than eliminate a viable residual reservoir. Chronic toxicity, acquired resistance, recurrence after treatment withdrawal, and uncertainty regarding patient selection must therefore be considered in preclinical and clinical development.

### 4.2 State Modulation Followed by Biomarker‐Defined Treatment

The “shock‐and‐kill” concept was originally developed in the context of human immunodeficiency virus latency, in which latent virus is first induced and then removed through subsequent clearance mechanisms [88]. In solid tumors, deliberate awakening could accelerate outgrowth if clearance is incomplete. The more defensible concept is therefore state modulation to increase therapeutic susceptibility, followed by a predefined biomarker‐matched treatment, rather than controlled awakening followed by assumed selective elimination.

At present, there is insufficient primary evidence to conclude that decitabine, azacitidine, or panobinostat function as bona fide dormancy‐reversing agents in ovarian or cervical cancer. What is better supported in gynecologic malignancies is the idea that epigenetic modulation can increase immune visibility, restore antigen presentation, and reshape the local tumor microenvironment. For example, Moufarrij et al. showed in ovarian cancer models that combined DNA methyltransferase (DNMT) inhibition and histone deacetylase 6 (HDAC6) inhibition enhanced antitumor immune signaling, increased interferon‐γ (IFN‐γ)‐positive CD8^+^ T cells, natural killer (NK) cells, and natural killer T (NKT) cells, and reduced MDSCs together with tumor burden [89]. These findings support the view that epigenetic intervention can alter tumor state and improve immune accessibility, even though they do not directly establish a true dormancy‐reversal effect. Additional studies in gynecologic cancer also suggest that epigenetic drugs may enhance the efficacy of subsequent treatment. Wilson et al. reported that in homologous recombination‐proficient ovarian cancer models, panobinostat altered chromatin state, enhanced the activity of olaparib, and was accompanied by changes in the tumor immune microenvironment [90]. Together, these studies support epigenetic immune remodeling and treatment sensitization, but do not demonstrate controlled awakening or subsequent clearance of a defined dormant‐cell population.

The “kill” phase is more directly supported by emerging gynecologic cancer studies identifying exploitable liabilities such as ferroptosis susceptibility, redox‐buffering dependence, and mitochondrial metabolic dependence. In BRCA1‐deficient ovarian cancer, Xie et al. demonstrated that PARPi can themselves induce ferroptosis, and that additional suppression of GPX4‐mediated ferroptosis protection markedly increases lipid peroxidation and tumor cell death in patient‐derived organoids and xenograft models [56]. This supports a biomarker‐defined interaction between PARP inhibition and ferroptosis vulnerability, but does not demonstrate selective identification or elimination of dormant MRD.

Accordingly, the most defensible interpretation at present is that “shock‐and‐kill” in gynecologic malignancies should not be regarded as a mature two‐step treatment paradigm. State modulation should remain in preclinical development and, only where stringent prerequisites are met, be evaluated in carefully controlled trials with a predefined clearance treatment, intensive monitoring, stopping rules, and rescue therapy. Principal risks include incomplete clearance, accelerated growth of surviving cells, selection of alternative resistant states, treatment toxicity, and heterogeneous responses among residual‐cell populations.

### 4.3 Decision Constraints and Requirements for Translation

The divergence between suppression of reactivation and state modulation creates a central clinical decision problem. Although current biomarkers can detect molecular residual disease and stratify recurrence risk, no biomarker reviewed here has been clinically validated to distinguish dormant, awakening, and actively proliferating residual‐cell states or to determine whether growth restraint should be preserved or the residual‐cell state should be therapeutically modified.

Based on the liquid‐biopsy evidence reviewed in Section 5.2, currently available assays support MRD detection and recurrence‐risk stratification rather than validated dormancy‐state assignment or treatment‐strategy selection. Accordingly, circulating tumor DNA (ctDNA) positivity or conversion alone cannot determine whether growth restraint should be preserved, state modulation should be attempted, or treatment should be escalated.

Translation of either dormancy‐directed concept will require an operational definition and validated biomarker of the targeted residual‐cell state; evidence that the intervention modifies that state rather than producing nonspecific tumor‐growth inhibition; an effective clearance mechanism for state‐modulation approaches; longitudinal monitoring for incomplete response, pathway escape, or accelerated progression; predefined stopping and rescue criteria; and prospective comparison with standard surveillance or established maintenance treatment. Conceptually, suppression of reactivation would require evidence of a viable, growth‐restrained residual‐cell state with identifiable reactivation signals, particularly when no reliable clearance strategy is available. In contrast, state modulation should be considered only when the targeted state can be modified predictably and followed by an effective, biomarker‐matched clearance treatment. These conditions remain proposed translational criteria rather than validated clinical decision rules.

Future studies should initially evaluate suppression of reactivation and state modulation as separate investigational concepts, using disease‐specific eligibility criteria, prospectively defined residual‐state biomarkers, and explicit safety endpoints for incomplete clearance or accelerated progression. Until these prerequisites are met, clinical decisions should remain anchored to established disease‐specific standards of care, and dormancy‐directed interventions should be restricted to preclinical development or carefully controlled biomarker‐guided trials. An evidence‐ and risk‐constrained overview of these therapeutic concepts is presented in Figure 3.

**FIGURE 3:**
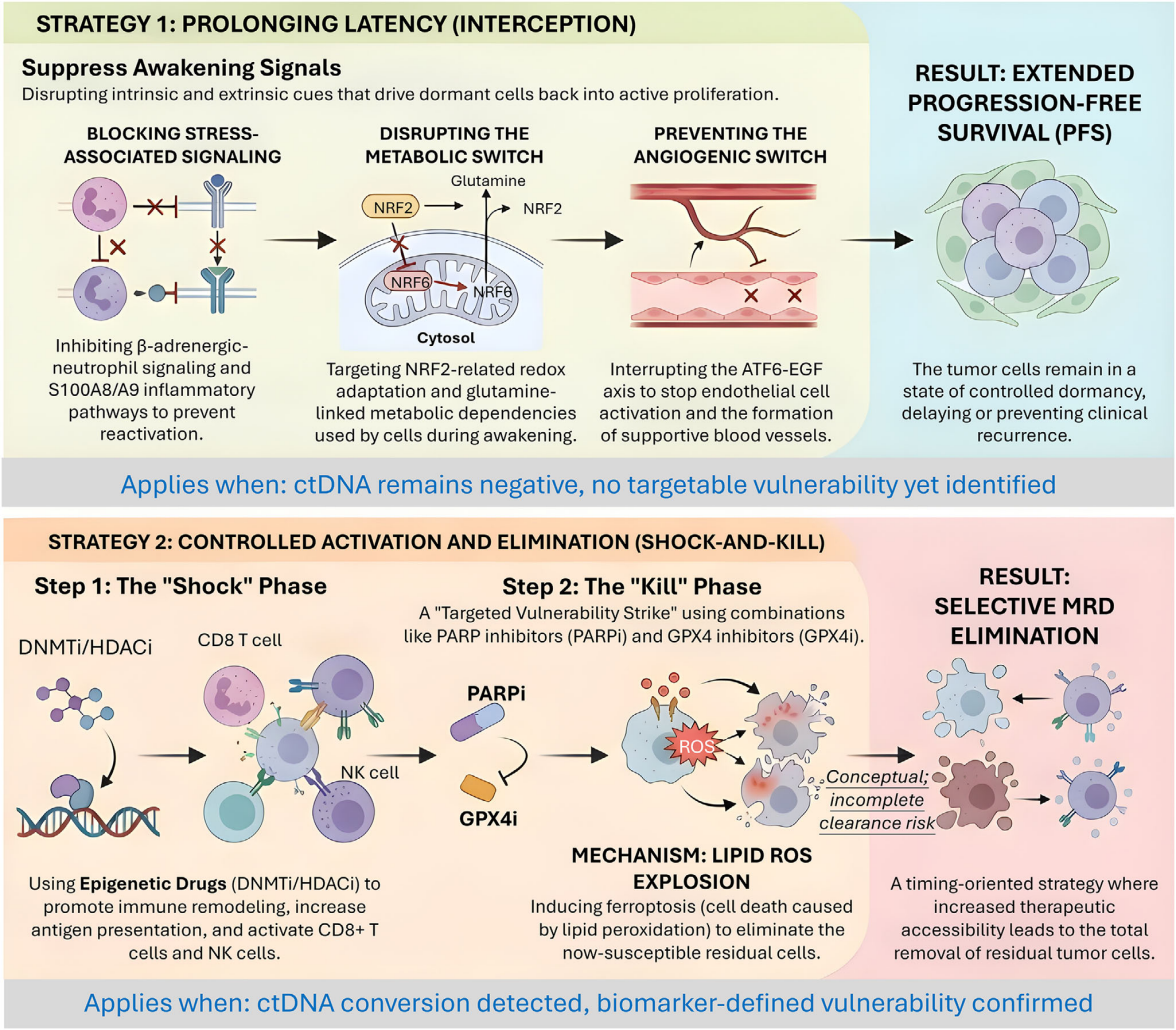
Therapeutic strategies for targeting tumor dormancy. Two distinct approaches are illustrated to manage dormant residual disease. Strategy 1: prolonging latency (Interception) aims to suppress awakening signals by disrupting the intrinsic and extrinsic cues that drive reactivation. This involves (i) blocking stress‐associated signaling (inhibiting β‐adrenergic‐neutrophil and S100A8/A9 pathways), (ii) disrupting the metabolic switch by targeting NRF2‐related redox adaptation and glutamine dependencies, and (iii) preventing the angiogenic switch via interruption of the ATF6‐EGF axis. These interventions aim to maintain cells in a state of controlled dormancy to extend progression‐free survival (PFS). Strategy 2: controlled activation and elimination (Shock‐and‐Kill) employs a sequential protocol. In the “Shock” phase, epigenetic drugs (DNMTi/HDACi) are utilized to promote immune remodeling and activate CD8+ T cells and NK cells. In the subsequent “Kill” phase, a targeted vulnerability strike using PARP inhibitors (PARPi) and GPX4 inhibitors (GPX4i) is applied. The primary mechanism involves a lipid ROS explosion, which induces ferroptosis to achieve selective MRD (minimal residual disease) elimination. Transition between strategies is guided by longitudinal ctDNA dynamics and biomarker‐confirmed vulnerability status; these criteria remain hypothesis‐generating and require prospective clinical validation.

## 5 Technologies for Characterizing Dormancy‐Associated Residual Disease

Translating dormancy‐directed strategies into clinical practice requires tools that can define both the biological state of residual disease and the timing of intervention. However, current platforms provide complementary but incomplete information and do not yet support validated dormancy‐state classification or treatment assignment. In this context, spatial multi‐omics and longitudinal liquid biopsy provide complementary forms of translational information. Spatial profiling helps identify candidate cellular ecosystems and local microenvironmental barriers within tumor tissue, whereas liquid biopsy offers a practical means of tracking MRD dynamics and recurrence‐risk changes over time. Together, these approaches provide a framework for biological stratification and longitudinal risk assessment, while their integration remains investigational for residual‐state inference and biomarker‐guided clinical‐trial development.

### 5.1 Spatial and Single‐Cell Profiling of Residual‐Cell Ecosystems

To advance dormancy‐associated residual‐disease research toward clinical translation, a central challenge is to identify and spatially localize candidate residual tumor‐cell states associated with long‐term persistence or recurrence within complex tissues and to characterize the surrounding immune, stromal, endothelial, and extracellular‐matrix features that may support or constrain these states. Although single‐cell RNA sequencing provides high‐resolution information on cell states, the main advantage of spatial multi‐omics lies in preserving the relative positions of cells within tissue architecture. This makes it well suited to identifying candidate residual‐disease niches and microenvironmental conditions associated with persistence, including local immune exclusion, stromal remodeling, abnormal endothelial barriers, and vascular‐associated supportive signals.

In ovarian cancer, Yeh et al. integrated spatial transcriptomics, genotype, and functional perturbation data and showed that high‐grade serous ovarian cancers contain genotype‐associated malignant cell states that predict the degree of T‐cell and NK‐cell infiltration, as well as immune‐evasive behavior. Their study also showed that manipulation of these malignant cell‐state regulators increased tumor‐cell susceptibility to T/NK‐cell killing, indicating that spatial maps are not only descriptive but can also identify experimentally testable candidate immune‐evasion nodes [91]. In addition, Kader et al. used multimodal spatial analysis to examine progression from fallopian tube precursor lesions to HGSOC and found that the local microenvironment shifts from early immune surveillance to immune suppression, accompanied by persistent interferon signaling, human leukocyte antigen E (HLA‐E)/major histocompatibility complex class I expression, and stromal remodeling [92]. In ovarian clear cell carcinoma, Tai et al. further showed that different recurrence groups display distinct immune‐like, immune signal‐high, and fibronectin‐high spatial patterns, supporting marked microenvironmental heterogeneity across ovarian cancer histotypes [41].

In endometrial cancer, Yu et al. combined single‐cell RNA sequencing with spatial transcriptomics and found that tumor epithelial cells can “educate” endothelial cells through midkine–nucleolin signaling, thereby shaping a less immune‐active microenvironment. Spatial proximity analysis supported the presence of this tumor‐endothelial interaction, suggesting that endothelial cells and adjacent stroma are not passive background components but active participants in the niche that supports residual disease [93, 94, 95]. In mismatch repair‐deficient endometrial cancer, high‐plex spatial transcriptomic profiling further identified an HLA class I/ DNA methyltransferase 3 alpha /CD8 regulatory axis associated with hot versus cold immune states, linking epigenetic regulation, antigen presentation, and local T‐cell infiltration [94]. In addition, integrated single‐cell and spatial analyses from anti‐programmed cell death protein 1 (PD‐1)‐treated endometrial cancer cases showed that therapeutic response is closely associated with local tumor microenvironment composition, supporting the use of spatial profiling to define tissue‐level features associated with immunotherapy response [95].

In cervical cancer, Wu et al. applied single‐cell and spatial transcriptomic approaches to compare adenocarcinoma and squamous cell carcinoma and found substantial differences in stromal and immune architecture. Their analyses identified endothelial subsets characterized by CXCL12/CCL2 signaling, distinct fibroblast states, including cancer‐associated fibroblast (CAF) subsets, and different macrophage polarization patterns [45, 96]. Lin et al. further focused on cervical cancer fibroblasts and identified CAF subsets with immunoregulatory functions. Functional analyses suggested that these stromal cells contribute not only to tumor proliferation and invasion but also to the establishment of an immunosuppressive microenvironment [96]. Together, these findings indicate that even within cervical cancer, different histologic subtypes and stromal compositions may have fundamentally different immune barriers and microenvironmental dependencies. These observations support disease‐ and histology‐specific biological stratification, but spatial findings alone should not be interpreted as validated treatment‐selection criteria. Within the entry–maintenance–reactivation framework, spatially localized low‐proliferative or stress‐adaptive states within immune‐protected, hypoxic, or matrix‐supported niches may generate testable hypotheses related to dormancy entry or maintenance. Inflammatory recruitment, angiogenic remodeling, extracellular‐matrix reorganization, and increased proliferative signaling may likewise nominate conditions associated with residual‐tumor outgrowth. These assignments remain hypotheses because most current studies analyze primary, metastatic, recurrent, or treatment‐exposed tumors at isolated time points rather than tracking a defined residual‐cell population through a dormancy‐to‐reactivation transition.

Taken together, current primary evidence in gynecologic malignancies supports the view that spatial multi‐omics can define immune‐excluded regions, abnormal endothelial barriers, stromal remodeling zones, and high‐risk tumor ecological niches, thereby providing a tissue‐level map for mechanistic stratification and prioritization of candidate microenvironmental targets. In the translational setting of dormancy‐directed management, a reasonable approach may therefore be to use spatial maps first to identify local barriers that restrict T‐cell entry, promote immune suppression, or protect residual cells, and then nominate these features for functional testing or clinical‐trial stratification rather than directly determining treatment sequence. At the same time, spatial multi‐omics is better suited to defining intralesional ecological states and intervention targets than to serving as a standalone platform for longitudinal monitoring of minimal residual disease. To convert spatial state characterization into dynamic residual‐disease assessment, it will likely need to be integrated with longitudinal liquid biopsy approaches based on repeated sampling.

Emerging platforms, including single‐cell multi‐omics, spatial proteomics, imaging mass cytometry, and multiplex immunofluorescence, may improve simultaneous assessment of chromatin regulation, signaling activity, proliferative status, and cell–cell interactions [44, 70]. Their current value lies primarily in biological stratification, identification of candidate niches, and generation of experimentally testable signatures; they remain experimental for routine dormancy‐phase classification or treatment assignment.

### 5.2 Liquid Biopsy for Dynamic MRD Monitoring and Recurrence‐Risk Stratification

Whereas spatial multi‐omics is well suited to characterizing intralesional ecological states and candidate residual‐disease niches, the main value of longitudinal liquid biopsy lies in its ability to enable repeated sampling during postoperative or post‐curative follow‐up, thereby allowing dynamic monitoring of minimal residual disease and detection of molecular signals that may precede radiographic relapse. Across multiple solid tumors, ctDNA has been shown to predict recurrence risk and therefore represents a promising tool for risk stratification and early identification of molecular recurrence, although ctDNA dynamics do not independently define the biological state of residual cells or the timing of dormant‐cell awakening.

In lung cancer, the LUCID study used the personalized, tumor‐informed RaDaR platform to monitor 88 patients with early‐stage non‐small‐cell lung cancer treated with curative intent. ctDNA detection at landmark post‐treatment time points was associated with a substantially increased risk of subsequent relapse and worse overall survival. In addition, ctDNA detection preceded radiographic confirmation of relapse by a mean of about 212.5 days, indicating that liquid biopsy can provide clinically meaningful lead time [97]. Related work from the TRACERx study (tracking non‐small cell lung cancer evolution through therapy [Rx]) further supported this principle, showing that tumor‐informed ctDNA dynamics can indicate recurrence risk before standard imaging and support postoperative molecular residual‐disease detection and risk stratification [98]. Taken together, these cross‐cancer studies suggest that the translational value of ctDNA lies not only in earlier recognition of recurrence, but also in identifying a clinically relevant interval between molecular disease detection and overt radiographic progression, rather than a direct window of biological reactivation.

In gynecologic malignancies, one of the clearest current examples comes from human papillomavirus (HPV) ctDNA in cervical cancer. In a multicenter study of 70 patients with stage IB‐IVA HPV‐positive cervical cancer treated with definitive chemoradiation, Han et al. measured HPV ctDNA at treatment completion, at 4–6 weeks, and at 3 months after therapy. Persistent detection of HPV ctDNA at any of these time points was associated with significantly worse 2‐year PFS. Patients who remained HPV ctDNA‐positive at 3 months had a 2‐year PFS of about 24%, compared with about 82% in those who were negative. The reported median lead time was about 5.9 months, supporting HPV ctDNA as a high‐value tool for MRD monitoring and early recurrence risk assessment in cervical cancer [99].

A transition from undetectable to detectable ctDNA may therefore identify a higher‐risk molecular recurrence window, but does not by itself establish dormant‐cell awakening. ctDNA positivity may reflect changes in tumor burden, proliferation, cell death, anatomical location, or DNA shedding, whereas ctDNA negativity does not confirm biological dormancy or exclude anatomically confined, low‐shedding, or very‐low‐volume residual disease. Accordingly, ctDNA status should not independently determine whether suppression of reactivation, state modulation, or treatment escalation is appropriate. A new positive or rising signal may instead prompt confirmatory imaging or tissue assessment, intensified surveillance, and consideration of eligibility for biomarker‐guided clinical trials, while treatment decisions remain based on established disease‐specific pathways, validated predictive biomarkers, and the overall clinical context.

### 5.3 Multimodal Integration for Residual‐State Inference and Validation

No single assay can simultaneously define the anatomical location, proliferative state, immune context, survival program, and therapeutic susceptibility of residual disease. Multimodal approaches may therefore integrate longitudinal liquid biopsy with spatial and single‐cell profiling, pathology imaging, circulating tumor‐cell analysis, extracellular‐vesicle biomarkers, methylation‐based assays, and clinical variables. Tissue‐based measurements provide local biological context, whereas blood‐based assays contribute temporal information through repeated sampling.

Artificial‐intelligence and machine‐learning methods may facilitate cross‐platform integration and improve recurrence‐risk stratification [100]. However, predictive performance alone does not establish biological dormancy, because a model trained on recurrence outcomes may identify high‐risk patients without distinguishing quiescent, reactivating, and actively growing residual disease. Clinical translation will therefore require standardized sampling, interpretable features, external validation, and prospective testing against longitudinal biological transitions and treatment outcomes. A clinically useful dormancy‐state biomarker should additionally identify viable residual disease, distinguish relevant residual‐cell states, demonstrate temporal reproducibility, and predict response to a defined intervention.

Taken together, spatial multi‐omics and longitudinal liquid biopsy provide complementary layers of translational information: the former defines high‐risk niches and local barriers within the tissue microenvironment, whereas the latter offers a practical means of tracking MRD dynamics and identifying windows for intervention over time. More precisely, spatial and single‐cell profiling characterize candidate residual‐cell states and local ecological conditions, whereas liquid biopsy enables scalable MRD monitoring and dynamic recurrence‐risk assessment. Their integration may support probabilistic residual‐state inference, but the resulting signatures should currently be regarded as candidate state‐associated or recurrence‐risk biomarkers rather than validated treatment‐selection tools. The complementary strengths, limitations, and translational roles of these two platforms are summarized in Table 3. Candidate biomarkers and translational readouts relevant to biological stratification, MRD monitoring, and future treatment‐selection research are summarized in Table 4. The integrated spatial and temporal translational framework is illustrated in Figure 4.

**TABLE 3:** Current roles and maturity of spatial, liquid‐biopsy, and multimodal platforms in MRD research.

Platform	Primary question	Major strength	Key limitation	Representative evidence/application	Current translational role
Spatial and single‐cell profiling	Which candidate residual‐cell states and niche features are present, and where?	Preserves tissue architecture and resolves cellular heterogeneity and interactions.	Usually cross‐sectional and tissue‐dependent; cannot establish longitudinal transitions alone.	HGSOC state/immune‐evasion mapping; endometrial tumor‐endothelial profiling; cervical stromal and histology‐specific ecosystems [41, 44, 45, 70, 91, 92, 93, 94, 95, 96].	Biological stratification and hypothesis generation; not validated for dormancy‐state or treatment assignment.
Longitudinal liquid biopsy	Is MRD detectable, and how does recurrence risk change over time?	Allows repeated sampling and may detect molecular disease before imaging.	Limited spatial context; sensitivity depends on shedding and assay depth; positivity does not define cell state.	Tumor‐informed ctDNA across solid tumors and HPV ctDNA after cervical chemoradiotherapy [97, 98, 99].	MRD monitoring and risk assessment; may prompt confirmation or trial consideration, but cannot select a dormancy‐directed strategy.
Integrated multimodal approach	Can tissue context and longitudinal signals improve probabilistic residual‐state inference?	Combines local ecological information with temporal MRD dynamics and clinical variables.	Requires standardized sampling, interpretable models, external validation, and prospective testing.	Future integration of spatial/single‐cell data, liquid biopsy, imaging, CTC/EV or methylation assays, and AI [100].	Framework for biomarker development and trial stratification; not a validated treatment‐selection system.

Abbreviations: AI, artificial intelligence; CTC, circulating tumor cell; ctDNA, circulating tumor DNA; EV, extracellular vesicle; HGSOC, high‐grade serous ovarian cancer; HPV, human papillomavirus; MRD, minimal residual disease.

**TABLE 4:** Candidate biomarkers and translational readouts for residual‐disease monitoring, biological stratification, and future validation.

Biomarker/readout	Signal captured	Specimen	Current maturity	Translational role and principal limitation
Tumor‐informed ctDNA MRD assays	Molecular residual disease and recurrence‐risk dynamics	Plasma	Clinically feasible in selected solid tumors; gynecologic implementation remains disease‐ and assay‐dependent	Supports post‐treatment MRD monitoring, risk stratification, confirmatory assessment, and trial eligibility [97, 98]; does not define dormancy state or independently select treatment.
HPV ctDNA	Persistent viral tumor signal and early recurrence risk in HPV‐positive cervical cancer	Plasma	Emerging clinically validated prognostic MRD readout in HPV‐positive cervical cancer [99]	Supports surveillance, intensified follow‐up, and prospective trial stratification; restricted to HPV‐positive disease and does not resolve residual‐cell state.
Emerging blood‐based readouts: CTCs, EV/exosome biomarkers, and methylation assays	Viable circulating cells; tumor/microenvironment‐derived cargo; tissue‐of‐origin and tumor‐associated epigenetic signals	Blood or other biofluids	Research‐stage to emerging; assay standardization and gynecologic MRD validation remain limited	May complement mutation‐based ctDNA and enable exploratory phenotyping or low‐shedding disease assessment; clinical thresholds and dormancy specificity are unvalidated.
Spatial and single‐cell state/niche signatures	Immune exclusion, endothelial/stromal barriers, malignant cell‐state regulators, and hypoxia/stemness programs	Tumor tissue	Research/preclinical	Supports biological stratification, candidate niche identification, and functional hypothesis generation [31, 32, 91, 92, 93, 94, 95, 96]; requires tissue and is not suitable for routine longitudinal monitoring.
Multimodal AI‐derived risk or state scores	Integrated pathology, molecular, spatial, liquid‐biopsy, and clinical features	Multimodal digital data	Research; mainly retrospective or prognostic [100]	May improve cross‐platform integration and recurrence‐risk stratification; requires interpretability, external validation, and prospective testing and does not establish biological dormancy state.

*Note*: Current maturity refers to feasibility for MRD or risk assessment, not to clinical validation as a dormancy‐state or treatment‐selection biomarker.

Abbreviations: AI, artificial intelligence; CTC, circulating tumor cell; ctDNA, circulating tumor DNA; EV, extracellular vesicle; HPV, human papillomavirus; MRD, minimal residual disease.

**FIGURE 4:**
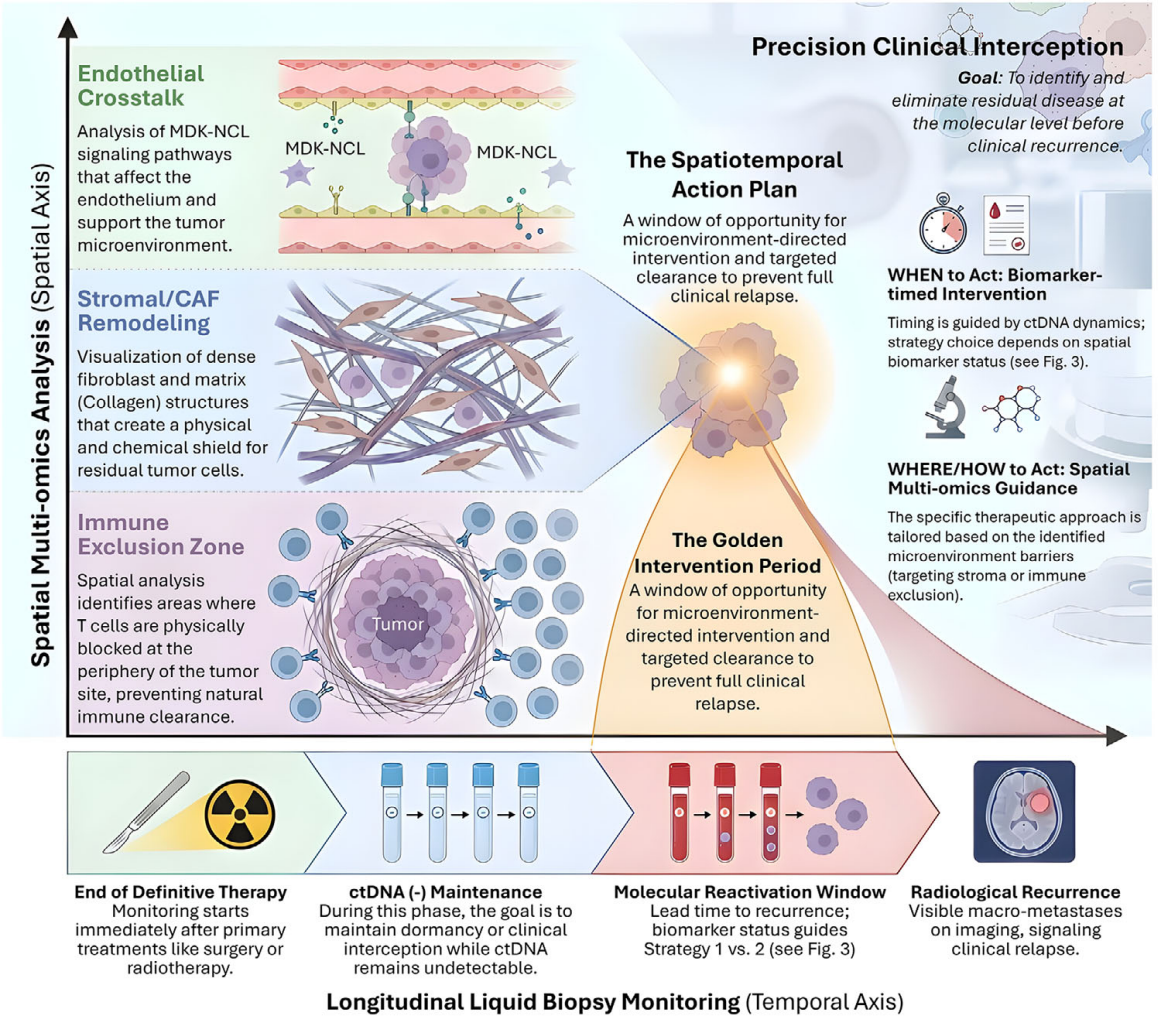
A spatiotemporal framework for precision clinical interception. This integrated model illustrates the synergy between longitudinal liquid biopsy monitoring (temporal axis) and spatial multi‐omics analysis (spatial axis) to define the golden intervention Period. On the temporal axis, monitoring begins at the end of definitive therapy, tracking the progression from ctDNA (−) maintenance to the molecular reactivation window—the critical “lead time” before clinical relapse. Simultaneously, the spatial axis identifies microenvironmental barriers to clearance, such as endothelial crosstalk, stromal/CAF remodeling (matrix shields), and immune exclusion zones. The spatiotemporal action plan leverages biomarker dynamics to determine the optimal timing (when to act) and spatial insights to tailor the therapeutic approach (where/how to act). By targeting residual disease during this molecular window, the strategy aims to achieve precision clinical interception, effectively eliminating the disease before it progresses to Radiological Recurrence, which is marked by visible macro‐metastases and clinical failure.

## 6 Conclusion and Future Translational Perspectives

Late recurrence remains a major challenge in gynecologic malignancies and is increasingly understood in relation to tumor dormancy and MRD. Current primary evidence supports the view that dormancy is not a passive state, but a dynamic process shaped by stress adaptation, metabolic rewiring, redox control, and sustained interaction with the immune and stromal microenvironment. However, direct evidence remains uneven across diseases: ovarian cancer currently provides the strongest preclinical support, whereas much of the endometrial and cervical cancer literature characterizes disseminated cells, treatment‐tolerant populations, stem‐like states, or recurrence‐associated microenvironments rather than bona fide dormancy [6, 7, 8, 10, 18, 19, 20, 22, 23]. Entry, maintenance, and reactivation should therefore be regarded as an organizing framework rather than a universally demonstrated sequence. These observations suggest that dormant or residual tumor cells should be regarded as an important biological focus for translational investigation rather than as already established dormancy‐specific therapeutic targets. In this context, candidate dependencies related to FAO, OXPHOS, redox buffering, ferroptosis resistance, mitochondrial plasticity, and adaptive stress‐response programs, together with epigenetic regulation, immune interactions, and extracellular‐matrix sensing, represent plausible points for intervention, although most remain investigational rather than established therapeutic vulnerabilities. The evidence reviewed here also supports a shift from treatment of overt relapse toward biomarker‐informed surveillance and clinical‐trial development before overt recurrence. The entry–maintenance–reactivation framework may help organize biological hypotheses, but it does not currently support routine phase‐specific treatment assignment. Suppression of reactivation and state modulation followed by treatment represent biologically distinct investigational concepts rather than inherently complementary strategies. Their clinical development will require disease‐specific patient selection, validated residual‐state biomarkers, appropriate clearance mechanisms for state‐modulation approaches, and explicit monitoring, stopping, and rescue strategies.

Achieving this will depend on better integration of mechanistic studies with translational tools that define residual‐disease biology and temporal recurrence risk without assuming that either type of evidence alone defines dormancy status or determines treatment choice. Spatial multi‐omics and single‐cell technologies may help characterize candidate residual‐cell states and local ecological constraints, whereas longitudinal liquid biopsy may support MRD monitoring and dynamic recurrence‐risk assessment. Multimodal integration with imaging, circulating tumor‐cell or extracellular‐vesicle assays, clinical variables, and artificial‐intelligence approaches may further improve residual‐state inference and patient stratification, but requires external and prospective validation [100]. Although major challenges remain, including biomarker stability, subtype‐specific heterogeneity, and validation of intervention timing, key priorities are to establish operational criteria for gynecologic tumor dormancy, validate state‐associated biomarkers and candidate therapeutic dependencies, define risk–benefit rules for the two intervention concepts, and conduct prospective MRD‐oriented trials. Tumor dormancy should currently be regarded as a biologically plausible but incompletely validated focus for biomarker‐guided translational investigation.

## Author Contributions

S.‐H.C. conceived and drafted the manuscript. S.‐H.C., R.‐W.P., C.‐C.W., C.‐W.L., and E.N.R. drew the figures, and discussed the concepts of the manuscript. Z.‐H.W. provided valuable discussion. T.‐H.‐H. and C.‐J.L. reviewed and edited of the manuscript. All authors have read and agreed to the published version of the manuscript.

## Funding

This research was funded by the National Science and Technology Council (114‐2628‐B‐075B‐001‐MY3), Taichung Veterans General Hospital (1143907B) and Kaohsiung Veterans General Hospital (KSVGH‐115‐056).

## Conflicts of Interest

The authors declare no conflicts of interest.

## Data Availability

Data sharing not applicable to this article as no datasets were generated or analyzed during the current study.
